# Morphological analysis of the sheathed flagellum of *Brucella melitensis*

**DOI:** 10.1186/1756-0500-3-333

**Published:** 2010-12-09

**Authors:** Jonathan Ferooz, Jean-Jacques Letesson

**Affiliations:** 1 Unité de Recherche en Biologie Moléculaire (URBM), Facultés Universitaires Notre-Dame de la Paix (FUNDP), 61 rue de Bruxelles, B-5000 Namur, Belgium; 2 GlaxoSmithKline Biologicals, 20 Avenue Fleming, B-1300 Wavre, Belgium

## Abstract

**Background:**

It was recently shown that *B. melitensis *is flagellated. However, the flagellar structure remains poorly described.

**Findings:**

We analyzed the structure of the polar sheathed flagellum of *B. melitensis *by TEM analysis and demonstrated that the Ryu staining is a good method to quickly visualize the flagellum by optical microscopy. The TEM analysis demonstrated that an extension of the outer membrane surrounds a filament ending by a club-like structure. The Δ*ftcR*, Δ*fliF*, Δ*flgE *and Δ*fliC *flagellar mutants still produce an empty sheath.

**Conclusions:**

Our results demonstrate that the flagellum of *B. melitensis *has the characteristics of the sheathed flagella. Our results also suggest that the flagellar sheath production is not directly linked to the flagellar structure assembly and is not regulated by the FtcR master regulator.

## Background

*Brucellae *are gram-negative, intracellular pathogenic bacteria that cause brucellosis in a variety of mammals, including humans. During a long time, they were considered as unflagellated. However, the presence of a sheathed flagellum has recently been discovered in *Brucella melitensis *[1,2].

The fact that the flagellum was never previously observed in *Brucella *is probably due to the very short period (the early exponential growth phase in rich liquid medium) during which the flagellum is produced. The availability of the genome sequences of *Brucella melitensis *and *Brucella suis *revealed the presence of all the flagellar genes needed for the construction of a functional flagellum, except the genes encoding the chemotactic system [3,4]. Today, the sequence of the genome of *B. abortus*, *B. ovis*, *B. canis *and *B. microti *are available, showing that flagellar genes are also present in these species [5-7]. Interestingly, the presence of mutations in several flagellar genes in the different *Brucella *species suggest that the flagellar regulon contains pseudogenes and that the filamentous appendage observed is a flagellum-derived structure [5].

The flagellum is a complex machine divided in three main structures [8]. The basal body structure is embedded in the membranes and functions like a tiny engine containing a motor, a stator and a secretion system. The hook structure is a universal joint transmitting the energy generated by the basal body to the filament. Finally, the filament is the most visible part of the flagellum and is made of about 20,000 monomers of flagellin with a length of several μm. The control of the flagellar assembly is a fine-tuned mechanism well described in *Salmonella enterica *serovar Typhimurium, *Escherichia coli *and *Caulobacter crescentus *(for reviews, see the references [9-12]).

It was recently described that flagellar regulation of *B. melitensis *was controlled by the FtcR flagellar master regulator which activates flagellar expression by binding directly on *fliF *promoter [13]. The mutation of *ftcR *decreases *fliF *expression and induces the extinction of the flagellin and hook proteins. The two LuxR-type regulators VjbR and BlxR are also required for flagellar activation [14,15]. VjbR is involved in the quorum sensing of *B. melitensis *and mediates the inhibitory effect of the *N*-dodecanoyl-_DL_-homoserine lactone (C12-HSL) [16]. In the early steps of the hierarchical flagellar regulation of *B. melitensis*, it was proposed that VjbR controls the expression of *ftcR *[13]. More recently, it was proposed that flagellar hierarchy is divided in three classes in *B. melitensis *(J. Ferooz *et al*., unpublished). In this later work, FlbT was described as the checkpoint regulator between the class II and III genes, and activates the flagellin production.

Here, we demonstrate that the extracellular appendage produced by *B. melitensis *is a flagellum with all characteristics of the sheathed flagella. Moreover, Δ*ftcR*, Δ*fliF*, Δ*flgE*, and Δ*fliC *mutants still produce a filament, probably an empty sheath regulated independently of the *ftcR *and *vjbR *flagellar pathway.

## Materials and methods

### Bacterial strains and culture conditions

All strains used in this study are listed in Table 1. All *Brucella *strains used in this study derive from *B. melitensis *16 M Nal^r ^(spontaneous nalidixic acid resistant mutant selected from *B. melitensis *16 M, received from A. Macmillan, Central Veterinary Laboratory, Weybridge, UK). The growth was measured by reading the optical density of the cultures at 600 nm. *B. melitensis *16 M growth curves in rich medium (1% yeast extract, 1.6% peptone, 0.5% NaCl) were performed from a late-exponential overnight culture obtained in liquid 2YT medium. *B. melitensis *16 M strains grew with shaking at 37°C in rich medium containing appropriate antibiotics from an initial OD_600 _of 0.05. *C. crescentus *CB15N grew in peptone-yeast extract (PYE complex media) at 30°C. Antibiotics were used at the following final concentrations: kanamycin, 50 μg ml^-1^; nalidixic acid, 25 μg ml^-1^.

**Table 1:** Bacterial strains

Strain*^a^*	Description	Source of reference
*B. melitensis *strains		
16 M Nal^r^	Spontaneous Nal^r ^strain of *B. melitensis *16 M obtained from A. P. MacMillan, Central Veterinary Laboratory, Weybridge, United Kingdom	Laboratory collection
*ftcR *mutant	*B. melitensis *16 M Δ*ftcR*::Kan^r^	[13]
*fliF *mutant	*B. melitensis *16 M Δ*fliF*::Kan^r^	Ferooz et al.*^b^*
*flgE *mutant	*B. melitensis *16 M Δ*flgE*::Kan^r^	Ferooz et al.*^b^*
*fliC *mutant	*B. melitensis *16 M Δ*fliC*::Kan^r^	Ferooz et al.*^b^*
*virB *mutant	*B. melitensis *16 M Δ*virB*	[22]
*C. crescentus *strain		
NA1000	*syn*-1000, synchronizable variant of strain CB15	[53]

*^a ^*Nal^r^, nalidixic acid resistant; Kan^r^, kanamycin resistant. *^b ^*Submitted for publication.

### Flagellum staining and visualization by phase-contrast microscopy

Bacterial flagella were stained using the "Ryu staining" method as described previously [17-19]. The Ryu stain has two components. Solution I (the mordant) contains 10 ml of 5% aqueous solution of phenol, 2 g of tannic acid, and 10 ml of saturated aqueous solution of aluminum potassium sulfate-12 hydrate. Solution II (the stain) is a saturated ethanolic solution of crystal violet (3 g in 25 ml of 95% ethanol). The final stain was prepared just before use by mixing 1 part of solution II with 10 parts of solution I and then by filtering the mixture through filter paper to remove coarse precipitate. A drop of cell culture was transferred onto the clean slide and covered with a cover slip. After 5 to 10 min, two drops of Ryu stain were applied to the edge of the cover slip and flowed under the cover slip by capillarity and mixed with the cell suspension. The cells were examined for flagella after 5 to 15 min at room temperature under a phase-contrast microscope. The edges of the cover slip on the slide were sealed with nail varnish.

### Visualization of the flagella by transmission electron microscopy (TEM)

The bacteria were grown in rich medium at 37°C to an OD_600 _of 0.25. Bacteria were centrifuged at 1000 r.p.m. for 20 min (Jouan), washed in PBS and fixed for 20 min in 50 μl of 4% paraformaldehyde pH 7.3. Samples were stored at 4°C. A drop (15 to 35 μl) of a solution of 1% Alcian blue was placed on a sheet of Parafilm. A carbon Formvar-coated grid was placed on a drop of solution for 5 min, carbon side down, washed five times in distilled water and then placed on a drop of bacterial suspension for 10 min on the same parafilm sheet. Grids with adherent cells were either (i) negatively stained for direct visualization on transmission electron microscope (TEM) or (ii) labeled with the anti-*Brucella *LPS O-chain, M epitope, mAb A156b3b2 [20,21] before staining as described previsously by Fretin *et al*.[1]. For immunolabeling, after 10 min on the drop of bacterial suspension, the carbon Formvar-coated grid was placed onto drops (15 to 35 μl) of the following reagents on the same parafilm sheet: 5 washes in phosphate-buffered saline (PBS)-glycine 5% (3 sec each), PBS-bovine serum albumin (BSA)(1 min), monoclonal antibody A156b3b2 diluted 1/20 in PBS-BSA 5% (1 h), five washes in PBS (10 sec each), rabbit anti-mouse immunoglobulin conjugated to ± 15 nm colloidal gold diluted 1/20 in PBS-BSA (1 h), three washes in PBS (10 sec each), 2 washes in distilled water (10 sec each). After a completed treatment protocol, the grid was negatively stained with 2% aqueous solution of uranyl acetate for 10 sec, the excess fluid was removed with a filter paper, and the grid was air dried. Specimens were examined using a Philips Technai 10 TEM. Note that protein A-colloidal gold were also used instead of rabbit anti-mouse immunoglobulin with the same results.

## Results

### The Ryu staining is a simple technique for the detection of the flagellum of *Brucella melitensis*

**Figure 1:**
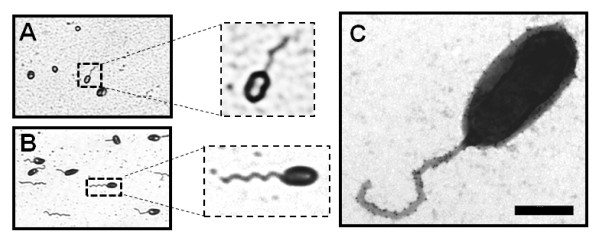
**Visualization of flagella**. Ryu staining of *B. melitensis *(A) *C. crescentus *(B) observed by phase-contrast microscopy. The samples were treated by the Ryu staining method as described in Materials and Methods. A flagellated bacterium is enlarged in a dotted square. (C) Negative-staining EM images of the sheathed polar flagellum of *B. melitensis *stained with uranyl acetate 2% and labeled with anti-LPS antibody conjugated to ± 15 nm gold particles. Bar, 500 nm.

Due to the short period of flagellar production (only at early exponential growth) and the low percentage of flagellated bacteria in *B. melitensis*, the visualization of the flagellum by TEM is difficult [1]. To easily detect the flagellated *Brucella*, we used the Ryu staining. An advantage of that technique is that the flagellum of *B. melitensis *can be visualized directly without any prior centrifugation (required for the TEM technique), minimizing the manipulation of the culture containing the pathogen. As positive control, we used *Caulobacter crescentus *because its flagellar expression is cell-cycle dependent and the flagella were previously visualized by Ryu staining [19]. We were able to visualize the flagellum of *B. melitensis *using the Ryu staining by phase-contrast microscopy (Figure 1A). Although the number of flagellated *B. melitensis *in the population is fewer than the flagellated *C. crescentus *(Figure 1B), the stained flagellum produced by *B. melitensis *is clearly detected by optical microscope. This result demonstrates that the Ryu staining is a simple technique allowing a rapid visualization of the flagellum of *B. melitensis *and with minimal sample manipulation.

### Ultrastructure analysis of the sheathed flagellum of *B. melitensis *by TEM

**Figure 2:**
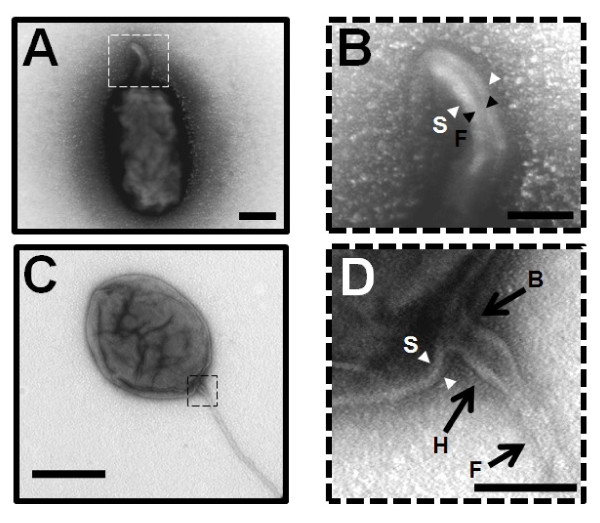
**Visualization of the flagellar sheath**. (A and B) Negative-staining EM images of the sheathed polar flagellum of *B. melitensis *stained with uranyl acetate 2%. (B)The central filament is indicated by black arrowheads with F and the sheath by white arrowheads with S. Bar, 200 nm. (C and D) Capture of the basal region of the flagellum. The image shows the basal body region (B with black arrow), the hook (H with black arrow) and is finished by the filament (F with black arrow). The structure is surrounded by a sheath extended from the outer membrane (S, white arrowheads). Bar, 100 nm. The images B and D are enlarged from the dotted square in image A and C respectively.

**Figure 3:**
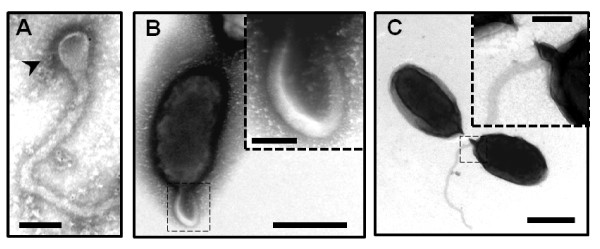
**Negative-staining EM images of the sheathed polar flagellum from *B. melitensis *stained with uranyl acetate 2%**. (A) The flagellum is ended by a club-like structure (black arrowhead). (B) Image of a curve structure at the pole of the cell. (C) Predivisional cells exhibited a flagellum at the septum division. A higher magnification of a part of flagellum (black square) is showing (dotted square). Bars, 500 nm in B and C; Bars, 100 nm (A, dotted squares in B and C).

We used the transmission electron miscroscopy (TEM) to analyze precisely the ultrastructure of the flagellum in *B. melitensis*. A sample of a *B. melitensis *culture at the early exponential growth phase in rich medium was stained with uranyl acetate 2% and visualized by TEM as described in the Materials and Methods section. We also labeled the bacteria with an anti-*Brucella *lipopolysaccharide (LPS) antibody and confirmed the presence of LPS on both the cell surface and the sheathed flagellum of *B. melitensis *(Figure 1C). *B. melitensis *produces a polar sheathed flagellum of about 50 nm diameter, showing an inner filament of about 11 nm diameter clearly surrounded by a sheath, which seems to be most likely a continuous extension of the bacterial outer membrane (Figure 1C and 2). As usually seen in other bacterial species producing a sheathed flagellum, the flagellum of *B. melitensis *is ending by a club-like structure (Figure 3A). A polar bended structure is also usually observed, like a nascent flagellum during the first steps of flagellar assembly (Figure 3B). This bended structure, and sometimes a flagellum, is visualized at the septum of division between two daughter cells, suggesting that flagellar assembly occurs at this site (Figure 3C). Taken together, these data clearly demonstrate that the appendage produces by *B. melitensis *has the typical features of the sheathed flagella and not a pilus-like structure or a remaining part of the flagellum.

### The sheath production is not dependent of the *ftcR *pathway and the flagellar structure

**Figure 4:**
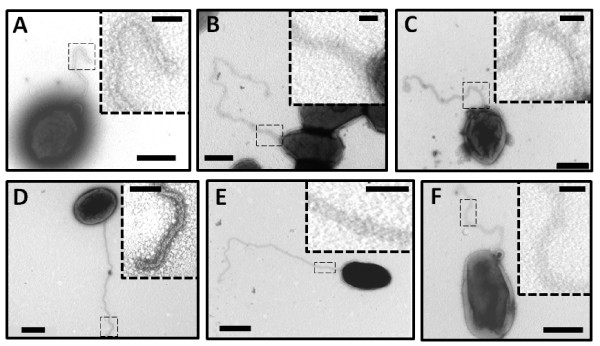
**Detection of a flagellum in *Brucella's *flagellar mutants**. Negative-staining EM images of the wild-type (D) compared to Δ*fliF *(A), Δ*flgE *(B), Δ*fliC *(C), Δ*virB *(E) and Δ*ftcR *(F) stained with uranyl acetate 2%. In dotted squares: higher magnification of a flagellum section. Bars, 500 nm and 100 nm in dotted squares.

To determine whether the mutation of flagellar genes affect flagellar assembly, Δ*fliF*, Δ*flgE *and Δ*fliC *mutants were analyzed by TEM. These mutants were created by gene replacement with the *aph*A4 cassette (J. Ferooz *et al*., unpublished). Surprisingly, these three mutants still produce a filamentous structure (Figure 4A, B and 4C). We previously demonstrated that FlgE and FliC protein cannot be detected by Western blot analysis in Δ*flgE *and Δ*fliC *respectively. Therefore, we propose that the appendage observed in Δ*fliF*, Δ*flgE *and Δ*fliC *is an empty sheath. The genome of *B. melitensis *contains the clusters of genes coding for only two extracellular structures: a type IV secretion system (T4SS) expressed at the stationary growth phase and a flagellum. In order to know whether the structure seen is a T4SS or not, a Δ*virB *mutant was visualized by TEM. This Δ*virB *mutant was made by removing the 12 open reading frames (ORFs) encoding the T4SS of *B. melitensis *16 M [22]. As seen in the wild-type strain (Figure 4D), Δ*virB *also produces a similar flagellar structure at the early exponential growth phase (Figure 4E). This result demonstrates that the extracellular appendage observed in flagellar mutants is not a T4SS.

The flagellar expression is not affected by mutation of the structural genes, indeed Δ*fliF *and Δ*flgE *still produce the flagellin FliC (J. Ferooz *et al*., unpublished). However, the flagellar master regulator FtcR is needed for *fliF *expression and the FlgE and FliC synthesis [13]. By intensively observing Δ*ftcR *by TEM, we also found some bacteria producing a filament-like structure, suggesting that sheath synthesis and assembly is likely independent of the FtcR pathway (Figure 4F).

## Discussion and Conclusions

Although it was demonstrated that *B. melitensis *produces a polar sheathed flagellum under specific conditions [1], there were still some doubts concerning the production of a classical flagellum by *Brucella *[5,23]. The conditions in which the flagellum is observed are not optimal because *Brucella *produces a flagellum only at the early exponential growth phase in rich medium [1]. Moreover, *Brucella *must be handled according to level 3 biosafety precautions [24], making difficult its manipulation for the preparation of sample for TEM analysis. This is the reason why we tested the Ryu staining technique, that allows an easier detection of flagellum than TEM as described in several flagellated bacteria such as *C. crescentus*, *L. monocytogenes, Bacillus subtilis *and *Salmonella Typhi *[19,25-27]. Interestingly, even with a low number of flagellated bacteria, this technique allows a quick and direct manipulation of the culture before the extinction of flagellar expression. In this work, we showed that the flagellum of *B. melitensis *can be easily visualized using the Ryu staining.

The structure of the flagellum produces by *B. melitensis *was analyzed by the TEM technique and several features of the sheathed flagella of other species were highlighted. At the present time, little is known about the formation, composition or function of flagellar sheaths in bacteria, and interestingly, *Brucella *is the only one rhizobiale to produce a sheathed flagellum [28,29]. Due to the sheath around the filament, the visible flagellum of *B. melitensis *has a diameter of 50 nm, which is larger than an unsheathed flagellum. However, the diameter of the bacterial filament is usually about 20 nm but we showed that the diameter of the inner filament in the sheath of *B. melitensis *is only 11 nm. This smaller diameter could be linked to the shorter amino acid sequence of the unique flagellin composed of only 282 amino acids in *B. melitensis*. In comparison, the flagellin sequence of *E. coli, S. enterica *serovar Typhimurium and *Pseudomonas aeruginosa *are between 488 and 498 amino acids.

Among the features of sheathed flagellum, the shape at the end of the flagella of *B. melitensis *is similar to a club-like structure, also described in other bacteria like *B. bacteriovorus*, *H. pylori *and *V. fischeri *[30-32]. This extension can be viewed as the continuity of the sheath at the end of the filament. The genome of *Brucella *lacks the FliD (or HAP2) homologue, a protein involved in the flagellin assembly at the top of the filament. One hypothetical function of this club-like structure could be the formation of a confined space facilitating the self-assembly of flagellin monomers into a filament, despite the lack of a FliD homologue. Similarly in *Vibrio alginolyticus*, it was assumed that the sheath could trap excreted flagellin to allow polymerization independently of FliD [33]. Secondly, the sheath of the flagellum of *B. melitensis *is likely an extension of the outer membrane and contains LPS, which is also observed in *H. pylori*, *B. bacteriovorus *and some *Vibrio *spp., [1,32,34-38]. Even if the sheath contains LPS, the sheath composition of *Vibrio *and *Helicobacter *is different from the outer membrane [30,39,40]. To the best of our knowledge, all these features have never been observed in a pilus-like structure, confirming that this appendage is a flagellum and not a pilus or flagellum-like structure.

Similarly to other α-proteobacteria, *Brucella *and *C. crescentus *are morphologically asymmetric [41]. *C. crescentus *couples flagellar biogenesis with cell cycle allowing the production of a flagellum at the swarmer pole of predivisional cell and a stalk at the other pole [11]. Surprisingly, we occasionally observed a bended structure or a flagellum at the septum division of *B. melitensis *rather than at the poles of predivisional cells. The localization of the flagellum at the septum of division is rarely observed in bacteria but is not unusual and is described in *B. bacteriovorus *and *Treponema phagedenis *[42,43].

In the last part of this work, we demonstrated that production of the sheath is probably unlinked to flagellar assembly in *B. melitensis*. Indeed, Δ*fliF*, Δ*flgE *and Δ*fliC *structural flagellar mutants still produce a filamentous appendage despite the absence of FlgE or FiC proteins. However, persistence of an empty sheath in flagellar mutants was often described in bacteria producing a sheathed flagellum as *Vibrio *species and *H. pylori *[44-48]. In *B. bacteriovorus*, deletion of flagellin *fliC3 *caused the synthesis of copious, disordered, tubular material resembling outer membrane [49,50]. The authors suggest that this structure is a disordered sheath without a normal flagellum inside. It is important to note that counting the number of flagellated bacteria in the population of the mutants in order to estimate a percentage of flagellation compared to the wild-type strain is not relevant due to the variability and the low number of flagellated bacteria detected between samples. Thus, it is possible that the population of flagellar mutants is less flagellated than the wild type population, but better conditions to enhance flagellation of *B. melitensis *will be needed.

In spite the fact that FtcR is the master regulator of flagellar assembly in *Brucella*, persistence of sheath production in Δ*ftcR *mutant also demonstrate that sheath expression is not dependent of FtcR and suggests that another unknown regulator is involved. Since the sheath surrounds the filament and is produced at one pole of the cell, even in flagellar mutants, it is possible that a flagellar pole marker coordinates both flagellum and sheath biogenesis at the cell pole before the FtcR activation. In *Vibrio alginolyticus *and *C. crescentus*, the localization of the flagellum at the pole is dictated by FlhF and PflI regulators respectively [51,52]. Polar localization of the pole marker PflI is independent of FliF, whose oligomerization into the MS-ring probably allows the definition of the site of flagellar synthesis, suggesting that PflI acts before or independently of this event [52]. Similarly, a regulator could couple flagellar assembly and sheath production independently of FtcR in *B. melitensis*.

Altogether, the data presented in this study proved that *B. melitensis *produces a flagellum with the characteristics of sheathed flagella described in other organisms. Flagellar assembly and sheath production are a complex regulatory mechanism that remains to be further investigated to gain a better understanding of the flagellum's function during *Brucella*'s infection.

## Competing interests

The authors declare that they have no competing interests.

## Authors' contributions

JF and JJL designed the experiments and interpreted the data. JF performed the experiments. JF wrote the manuscript. All authors read and approved the final manuscript.
